# Determination of Punicalagins Content, Metal Chelating, and Antioxidant Properties of Edible Pomegranate (*Punica granatum L*) Peels and Seeds Grown in Morocco

**DOI:** 10.1155/2020/8885889

**Published:** 2020-09-17

**Authors:** Talal Sabraoui, Taleb Khider, Boubker Nasser, Rabiaa Eddoha, Abderrahman Moujahid, Maryam Benbachir, Abdelkhalid Essamadi

**Affiliations:** ^1^Faculty of Sciences and Technology Laboratory of Biochemistry & Neurosciences, Applied Biochemistry and Toxicology Team, Hassan First University, 26000 Settat, Morocco; ^2^Food Chemistry, Department of Chemistry and Pharmacy, Emil Fischer Center, Friedrich-Alexander Universitât Erlangen-Nürnberg (FAU), Nikolaus-Fiebiger-Straße 10, 91058, Germany

## Abstract

Pomegranate (*Punica granatum L*) is widely cultivated in the Mediterranean countries especially in Morocco. Pomegranate peel and seed contain considerable amounts of phenolic compounds with antioxidant activity. The aim of the present study was to phytochemically characterize the pomegranate peels and seeds obtained from three Moroccan provinces, using UHPLC-DAD. In addition, total phenolic content (TPC), total flavonoid contents (TFC), and metal chelating of pomegranate peel were also evaluated. The results showed that pomegranate peel possesses the highest phenolic (TPC: 224.39 mg GAE/g dw) and flavonoid (TFC: 62.64 mg rutin/g dw) contents. Punicalagin-*β* and punicalagin-*α*, are the abundant compounds found in peel: 216.36 ± 9.94 mg/g, 154.94 ± 5.21 mg/g, respectively. Pomegranate peels showed significantly (*p* < 0.05) high antioxidant activity 1-diphenyl-2-picrylhydrazyl (DPPH) EC_50_: 42.71 ± 0.04 *μ*g/mL, 2.2′-Azino-bis(3-Ethylbenzothiazoline-6-Sulfonic Acid) (ABTS) EC_50_: 62.15 ± 0.01 *μ*g/mL), and chelating activity (FRAP 1.85 ± 0.00 mg ascorbic acid equivalents/100 g, Fe^2+^: 2.52 ± 0.01 *μ*mol EDTA equivalents/g dw) compared to seeds. A positive correlation between antioxidant activity and total phenolic was found. According to achieved results, high antioxidant capacity of pomegranate extracts, especially peel, shed light to further use as natural food preservatives. Pomegranate peel could be used for the fortification of food with fiber by introducing it in dietary, as well as in health applications due to its higher antioxidant capacity.

## 1. Introduction

Pomegranate (*Punica granatum*. *L*) is a highly appreciated edible fruit tree in the world. The source of pomegranate is related to central Asia, from Iran to China and Eastern India [1]. Nevertheless, the cultivation of the fruit has spread to Mediterranean countries like Turkey and North Africa. Presently, the commercial cultivation of pomegranate is mainly concentrated in the Mediterranean countries followed by Asian countries [2]. Different cultures as ancient Egyptians and Indians used pomegranate in traditional medicine for many centuries [3].

In Morocco, dried peel of pomegranate is still used until today as a natural traditional treatment of diarrhea and dental plaque. The therapeutic potential of pomegranate has pushed both consumers and researchers to increase the interest in pomegranate bioactive compounds. Indeed, many studies have been conducted in the last few decades on the phytochemicals contents and antioxidant potential of pomegranate [1, 4–11]. Due to its sweet flavor, arils are the most edible part of pomegranate. However, data from literature demonstrated that the pomegranate peel had the highest antioxidant capacity comparing to seed and juice [12, 13]. Today it is accepted that pomegranate peel demonstrates good pharmacological properties, such as antioxidative [13], anticancer [14], and antidiabetic [15]. The comestible part of the fruit is particularly the arils. Considered a waste products, peel could have useful applications in meat conservation [16] and food industry [17]. Phenolic compounds as anthocyanins, punicalagin, and ellagic acid can represent about 50% of the fruit [9]. In fact, the peel is considered a good natural source of phenolic compounds [10], many studies have shown a high correlation between the antioxidant activity and amounts of phenolic compounds [12]. Pomegranate seed represented about 20% of the fruit and demonstrated interesting therapeutic applications and a good source biocompounds and antioxidants [18]. A study by Shaban et al. [19] revealed that the administration of pomegranate seed oil showed a significant protective effect against diethylnitrosamine (DEN) induced oxidative stress and apoptosis in hepatocytes. The benefits of pomegranate seed are mainly associated with the content of conjugated linolenic acids [20, 21].

Moroccan production of pomegranate overreaches 58,000 tones in a total area of 4,625 hectares; Tadla Azilal is the leading region in Morocco with a production of 28,000 t in an area of 1,410 hectares [21]. Studies on Moroccan pomegranate fruit have focused on genetic diversity [22], or physicochemical characteristics, total phenolic, and the antioxidants of juice [23, 24]. To the best of our knowledge, this is the first study characterizing the phenolic compounds and antioxidants activity of the Moroccan pomegranate peel and seed extracts grown in three distinct provinces: Beni Mellal, Settat, and Berkane. Given the wide spectrum of health-promoting activities of pomegranate peel, this work aimed to prepare methanolic fractions from peels and seeds of pomegranate, evaluate their antioxidant activity, and establish a correlation between phenolic compounds and antioxidants capacity.

## 2. Materials and Methods

### 2.1. Chemicals

All chemicals and reagents were purchased from Sigma-Aldrich chemistry (Germany); punicalagin (≥98%), ellagic acid (≥98%), and gallic acid (97.5%) were purchased from Sigma-Aldrich (Taufkirchen, Germany).

### 2.2. Fruit Samples and Extraction Procedures

Mature pomegranate fruits, variety “SEFRI”, were purchased from three geographical origins Beni Mellal, Settat, and Berkane provinces of Morocco. Fruits were manually peeled, collected peels and seeds were rinsed with distilled water and dried in an oven (Binder, BD 56 Germany) with air circulation at 40°C. Samples were ground in a laboratory grinder and passed through a sieve of 0.25 *μ*m (Retsch, Germany). The dried sample was then stored at 20°C until further use. The peel and seed powder (10 g) were extracted with 60 mL of methanol by magnetic stirring at room temperature for 24 hours. The extracts were filtered using Whatman no.41 filter paper. The residue was reextracted with 50 mL of methanol and filtered. The pool of extracts were concentrated under vacuum at 40°C (rotary evaporator Buchi R-210, Switzerland) [19].

### 2.3. Total Phenols and Flavonoid Content

The total phenolic content was determined according to the method of Folinc-Ciocalteu [25]. Briefly, pomegranate extracts (0.2 mg) were mixed with 0.1 mL of 10-fold diluted Folin-Ciocalteu reagent. After 5 min, 0.8 mL of sodium carbonate 7.5% (*w*/*v*) solution was added to the mixture and incubated for 30 min at 30°C then absorbance of the solution was read at 765 nm using Genesys-5 UV-visible spectrophotometer. Results were expressed as mg of gallic acid equivalents (GAE) per gram of dry weight (dw). The determination of flavonoid content is based on the formation of the complex flavonoid and aluminum [26]. Briefly, 0.5 mL of both peel and seed extract was mixed 0.5 mL aluminum chloride 2%, then 3 mL of potassium acetate 5% (Merck, Germany) was added, after 40 min at room temperature, and the absorbance was measured at 415 nm. Results are expressed as rutin equivalent in mg/g.

### 2.4. UHPLC-DAD Quantitative Analysis

Quantification of phenolic compounds was performed using ultrahigh-performance liquid chromatography equipped with hyphenated diode array detection (UHPLC-DAD; Thermofisher, Idstein, Germany). The detection wavelengths of 280 nm and 520 nm were selected. A HSS-T3 1.7 *μ*m column (50 mm × 2.1 mm) (Waters, USA) was used. 10 *μ*L of sample was injected, and 22°C temperature of column was ensured throughout the analytical process. Eluent A consisted of 0.1% formic acid in MilliQ water and eluent B (methanol). The flow rate was 0.3 mL/min. The gradient was optimized as follows: 1-5 minutes 1-5% B; 5-10 minutes 5-25% B; 10-13 minutes 25-95% B; 13-16 minutes 95% B. The standard polyphenols: gallic acid, (*α*,*β*)-punicalagin, and ellagic acid were mixed and dissolved in methanol to concentrations of 60 *μ*g/mL, 1000 *μ*g/mL, and 300 *μ*g/mL, respectively. Then, the phenolic compounds were quantified using the UHPLC Chromeleon software. For this purpose, calibration curves were prepared from gallic acid: 0.03-60 *μ*g/mL; *r*^2^ = 0.9988; ellagic acid: 0.03-300 *μ*g/mL; *r*^2^ = 0.999, punicalagin-*α*: 0.16-1000 *μ*g/mL; *r*^2^ = 0.9995 and punicalagin-*β*: 0.16-1000 *μ*g/mL; *r*^2^ = 0.9992 [27].

### 2.5. DPPH Assay

DPPH assay was measured according to the method described by Singh et al. [25]. Different concentrations of pomegranate peel and seed extracts were placed in different tubes, 5 mL of a 0.1 mM methanolic solution of DPPH was added to 100 *μ*L of every extract, and then, the tubes were shaken vigorously. A positive control (methanol instead of the sample) was prepared, and methanol only was used to zero the spectrophotometer. The tubes were allowed to stand at room temperature for about 30 min. The change of absorbance was measured at 517 nm. Radical scavenging capacity was estimated as percent of DPPH and expresses as a function of the sample concentration using the following formula: radical scavenging activity (%) = (control OD − sample OD/control OD) × 100. The antioxidant activity was calculated as the effective concentration of a sample required to decrease the absorbance of the positive control by 50% (EC_50_).

### 2.6. ABTS Assay

ABTS assay was based on the method of Ramos et al. [28] with slight modifications. Briefly, the radical ABTS solution was prepared by mixing equal volume of ABTS stock solution 7 mM with 2.45 mM of potassium persulfate. The mixture was allowed to stand for 16 hours at room temperature in the dark. Before been used, different concentrations of this solution in acetate buffer (pH 4.6 50 mM) were prepared to obtain an absorbance of 0.700 ± 0.02 at 734 nm. The assay was performed in a 96-well microplate. Hence, 150 *μ*L of the diluted sample was added in the plate followed by adding 150 *μ*L of the ABTS solution. Absorbance was measured after 6 min by spectrophotometer (Synergy HT, Bio-Tek Instruments, Winooski, VT, USA). ABTS scavenging percentage was calculated using the following equation: ABTS scavenging (%) = (control OD–sample OD/control OD) × 100. The EC_50_ values were calculated from standard curves.

### 2.7. FRAP Assay

Determination of the ferric reducing antioxidant power (FRAP) was based on the method by Oyaizu et al. [29]. 500 *μ*L of peel and seed was added to 1.25 mL of phosphate buffer (0.2 M, pH 6.6); 1.25 mL of ferricyanide potassium is added to each tube, followed by 1.25 mL of trichloro-acetic acid (10% *v*/*v*). The mixture is incubated at 50°C for 15 min and diluted in distilled water (1 : 2, *v*/*v*), and 0.25 mL of ferric chloride 1% was added. The absorbance was measured at 700 nm and results are expressed as equivalent mg ascorbic acid equivalent (AAE) per 100 g of DW.

### 2.8. Fe^2+^ Chelating Activity Assay

Pomegranate peel and seed extracts of ferrous ions Fe^2+^ were measured using the method described by Dinis et al. [30]. Briefly, 0.5 mL of extract at different concentrations was added to 1.6 mL of distilled water, and 0.05 mL of FeCl_2_ (2 mM) was added, followed by adding 0.1 mL Ferrozine (5 mM). The mixture is incubated at room temperature for 10 min, complex of Fe^2+^, and Ferrozine complex was measured at 562 nm. The chelating activity of the extract was calculated using the following equation: metal chelating activity (%) = (OD control − OD sample/OD control) × 100. The results were expressed as *μ*mol of EDTA equivalent/g of dry weight.

### 2.9. Statistical Analysis

Data were analyzed by the IBM SPSS V 23 software. One-way (ANOVA) and post hoc Tukey's HSD tests were used for multiple comparisons. Differences were considered significant at *p* < 0.05. Correlation values between experimental data were evaluated using Pearson's coefficient.

## 3. Results and Discussion

### 3.1. Total Phenolic and Total Flavonoid Content in Pomegranate Peels and Seeds

Pomegranate is one of the richest fruits with phenolic compounds, namely punicalagin, anthocyanins, and punicalin gallic and ellagic acids. Yet, a lack of information exists about the phenolic content of peel and seed of Moroccan pomegranate. Previous studies showed methanol as the solvent to achieve maximum yield of extract and maximum phenolic content from different parts of the fruit [30–32]. In our study, peel and seed extracts from pomegranate fruits grown in three different Morocco provinces were studied. The yields of methanolic extracts obtained in fruits from Beni Mellal, Settat, and Berkane provinces were 36%, 43%, and 53%; and 16%, 22%, 21% in peel and seed, respectively. These data are comparable to values found by Pagliarulo et al. [33] in peel using 50% ethanol/water (*v*/*v*), and higher than data reported by Singh et al. [25] when using methanol as solvent 9.38 and 8.62% in peel and seed, respectively. Differences in yields could be related to sample particle size. A study by Wang et al. [32] suggest that smaller sample particles size increased the extraction rate. The concentrations of total phenolic content found in Beni Mellal, Berkane, and Settat peel were 3.52, 3.29, and 3.30-folds higher than the content determined in seeds, respectively (Table 1). In fact, other works reported similar results [13, 34, 35], confirming that the highest phenolic compounds are in the pomegranate peel. Thus, the total peel polyphenols ranged from 204.59 to 224.39 mg GAE/g dw, slightly lower than those found by Li et al. [3]: 249.4 mg GAE/g dw of pomegranate peel extract in comparison with pomegranate pulp extract. Khalil et al. [36] studied the punicalagin contents and antioxidant activity after juice extraction obtaining a phenolic content of 289.40 mg GAE/g dw in peel methanolic extract. These variations in the values in the total phenolic content could be due to the varietal difference. Seed phenolic compounds were evaluated between 62.17 and 67.86 mg GAE/g dw, consistent with the data reported by Orak et al. [11] where the phenolic compounds of seed using methanol as a solvent ranged from 54.48 to 63.43 mg GAE/g. While, other study reported lower values, ranging from 1.29 to 2.17 mg GAE/g of China-pomegranate seeds, also for methanolic extracts [7].

In terms of total flavonoid contents found in Beni Mellal, Berkane, and Settat peel extracts, the values were 36, 24, and 25-fold higher, respectively, than the content in seeds (Table 1). According to our study, the total flavonoids ranged from 52.13 to 62.64 and 1.76 to 2.11 mg rutin/g dw in peel and seed, respectively; these values are higher than found by Orak et al. [11], where the content of flavonoids ranged from 14.37 to 20.52 and 5.92 to 16.92 rutin/g dw in peel and seed, respectively. It is noteworthy that Beni Mellal pomegranate peels extracts contained the highest levels in comparison to Settat and Berkane.

The obtained flavonoids contents data confirms that the pomegranate peel fraction possesses the highest phenolic compounds, which totally agree with previous studies [34, 37]. Furthermore, statistical mean values showed that there is no significant (*p* < 0.05) differences between the three regions in terms of phenolic and flavonoid contents.

### 3.2. HPLC Quantitative Analysis

**Figure 1:**
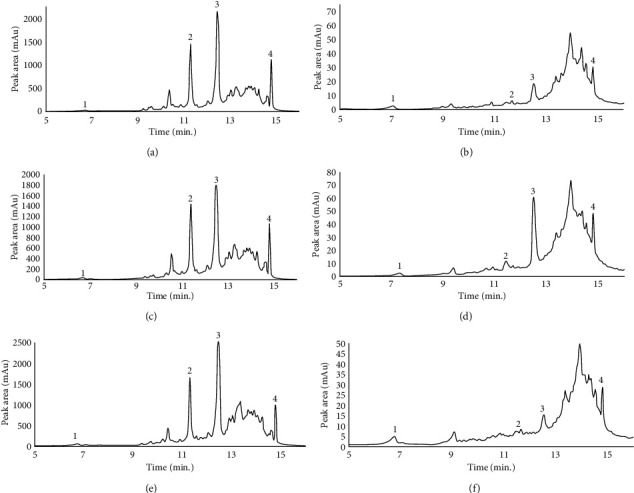
UHPLC-DAD chromatogram of the polyphenol extract of pomegranate peel powder ((a) Beni Mellal, (c) Settat, (e) Berkane) and pomegranate seed powder ((b) Beni Mellal, (d) Settat, (f) Berkane). Phenolic compounds were detected at 280 nm; the peaks were assigned to (1) gallic acid, (2) *α*-punicalagin, (3) *β*-punicalagin, and (4) ellagic acid.

The ellagitannins are considered the most abundant polyphenols in pomegranate peel as its content can reach 66% of the total polyphenols in the peel [38]. The characterisation of the phenolic compounds present in the methanolic pomegranate extracts was performed using UHPLC. The qualitative-quantitative analysis of punicalagin anomers, gallic acid, and ellagic acid demonstrated that the concentrations of punicalagin isomers, gallic acid, and ellagic acid were lower in the seed compared to peel (Table 1). Moreover, the chromatographic patterns of the phenolic fraction show dominance of punicalagin-*β* and punicalagin-*α* in relation to other phenolic compounds, in both peel and seed extract (Figure 1).

Accordingly, polyphenolic analyses showed higher punicalagin content than the ellagic acid and gallic acid content in the peel and seed for the three regions, representing about 6- to 100-fold increase in relation to the gallic acid content. The pomegranate concentration of punicalagin might varied between 39.8 and 121.5 mg/g dw [39], lower than our results that varied between 120.9 and 210.6 mg/g dw (Table 1). In another study conducted by Masci et al. [37], concentrations of selected phenolic compounds in peel were determined to be 11.85-63.61 mg/g for ellagic acid and 7.39–41.36 mg/g for punicalagin-*α* and punicalagin-*β*. It must be noticed that these authors used an extraction procedure with ethanol, which provided a lower starting ellagic acid yield in relation to methanol at room temperature [25].

### 3.3. Antioxidant Activity Assessment

The antioxidant activity of pomegranate extracts was evaluated by two different radicals' assays, DPPH and ABTS (Table 2). Peel extracts demonstrated the strongest radical scavenging activity in relation to seed extracts, also reported by other research groups [12, 13, 37]. Antioxidant activity evaluated by DPPH assay of Beni Mellal, Settat, and Berkane pomegranate peel were 2.98-, 2.04-, and 3.16-fold higher than the respective seeds. Furthermore, a strong correlation was found between the total phenolic content of peel and seed and their respective radical scavenging activity (Table 3).

Commonly known, pomegranate peel polyphenolic compounds contribute to its powerful antioxidant activity. In fact, an extensive screening of 1,000 herbal plant extracts by Niwano et al. [40] revealed that pomegranate peel exhibits the strongest antioxidant activity. Panichayupakarananta et al. [39] studied the ellagic acid content and antioxidant activity in pomegranate fruit peel and found an EC_50_ 38 *μ*g/mL in the methanolic extract, while in this work, the values varied between 42 and 65 *μ*g/mL in peel and 888 and 1,945 *μ*g/ml in seed. In contrast, a study by Liu et al. [41], evaluating the antioxidant properties of pulp and peel, showed that peel methanolic extract exhibits a lower scavenging activity (EC_50_ 12−25 mg/mL). Higher values (EC_50_ 0.84 mg/mL) were reported in comparison between edible and no edible pomegranates varieties in Tunisia [42]. A previous study by Khalil et al. [43] had also reported that fruit peel exhibited good scavenging activity using DPPH, their values varied between 63.36 ± 3.20 and 78.23 ± 4.11%.

Here, the antioxidant capacity by the ABTS method revealed lower antioxidant activity than that evaluated by the DPPH assay. Yet, the antioxidant activity of pomegranate peel was 6.5-fold higher than the one found in seed extracts (Table 2). These results are in agreement with those reported by Elfalleh et al. [44], with EC_50_ values of peel between 0.062 and 0.085 mg/mL and 4.03 and 4.83 mg/mL for seed extracts. Data from the ABTS assay showed a good correlation with the phenolic contents (*r* = 0.987, *n* = 3) (Table 3).

ABTS and DPPH. Radicals scavenging activity of pomegranate peel and seed is attributed to their capacity of donating hydrogen from their hydroxyl group, thus neutralising free radicals by forming stable complexes. Commonly known, pomegranate peel compounds contribute to its antioxidant activity; previous studies mentioned the strong correlation between antioxidants activity and phenolic contents [25, 42].

### 3.4. FRAP Assay

The ferric reducing antioxidant power (FRAP) is a colorimetric method used to evaluate the antioxidant activity, different from previous methods, as it does not involve any free radical. This methodology is based on the reduction of the complex Fe3^+^ and ferricyanide complex to the ferrous form. The reducing power values expressed as mg ascorbic acid equivalent (AAE)/g of dw of peel or seed are shown in (Table 2). The values varied from 185.56 to 251.02 mg AAE/100 g for peel, whereas seed showed valued between 0.12 and 0.20 mg AAE/100 g, confirming the higher antioxidant capacity of the peel.

### 3.5. Fe^+2^ Chelating Activity

Metal chelation could provide important antioxidative effect by retarding metal catalysed oxidation of reactive oxygen species. In fact, phenolic compounds exhibit chelating effect when they are deprotonated [40]. As seen in Table 2, the metal chelating metal capacity of peel was found higher than seed. The data exhibited that metal chelating capacity ranged between 1.401 and 2.525 *μ*mol EDTA equiv/g dry weight in peel extract, whereas no significant chelating effect was found in seed. This finding could be explained by the small amount of total flavonoids presents in seeds. The strong correlation between flavonoid amounts in food samples and chelating ability has been demonstrated in literature, as an example, the quercetin has three metal binding sites [40].

## 4. Conclusions

In conclusion, in this study, pomegranates from three different regions in Morocco were analyzed for antioxidant activity and phenolic contents in peel and seed upon methanolic extraction. In the peel, punicalagin were the predominant compounds; also, gallic and ellagic acids were detected, which contribute to the significant correlation with the antioxidant activity. However, no significant difference of phenolic compounds or antioxidant activity was found between regions. The achieved results, in particular the high antioxidant capacity of pomegranate peel, suggest further application as natural food preservatives and as a health supplement rich in natural antioxidants.

## Figures and Tables

**Table 1:** Qualitative–quantitative analyses of the polyphenolic fraction in selected extracts.

		Total phenolic (mg GAE/g)	Total flavonoids (mg RE/g)	Punicalagin-*β* (mg/g)	Punicalagin-*α* (mg/g)	Ellagic acid (mg/g)	Gallic acid (mg/g)
Peel	Settat	223.21 ± 15^a^	52.12 ± 1.36^a^	200.30 ± 5.29^a^	128.57 ± 2.74^a^	34.43 ± 0.91^a^	2.14 ± 0.00^a^
	Beni Mellal	224.39 ± 3^a^	62.63 ± 3.23^b^	200.21 ± 8.78^a^	130.66 ± 4.58^b^	35.00 ± 0.77^a^	1.86 ± 0.05^b^
	Berkane	204.58 ± 1.96^a^	46.17 ± 2.18^a^	216.36 ± 9.94^a^	154.94 ± 5.2^a^	32.14 ± 0.53^b^	2.23 ± 0.18^c^
Seed	Settat	63.34 ± 0.7^b^	2.11 ± 0.28^c^	1.75 ± 0.64^b^	2.93 ± 0.37^c^	1.60 ± 0.27^c^	0.14 ± 0.00^d^
	Beni Mellal	67.85 ± 1.98^b^	1.76 ± 0.02^c^	1.10 ± 0.4^b^	1.15 ± 0.12^d^	1.47 ± 0.21^c^	0.13 ± 0.00^e^
	Berkane	62.17 ± 3.26^b^	1.94 ± 0.00^c^	1.30 ± 0.27^b^	1.44 ± 0.03^e^	1.17 ± 0.04^c^	0.20 ± 0.04^f^

Different letters of within the same column is indicating significant differences at *p* < 0.05. All data are expressed as mean ± SD of at least three replicates of each sample. GAE: gallic acid equivalents; RE: rutin equivalents.

**Table 2:** Antioxidant activities of pomegranate peel and seed.

		DPPH assay EC_50_ (*μ*g/mL)	ABTS assay EC_50_ (*μ*g/mL)	FRAP assay mg AA/100 g dw	Fe^2+^ chelating activity (*μ*mol EDTA equiv/g dw)
Peel	Settat	43.13 ± 0.06^a^	62.15 ± 0.01^a^	1.47 ± 0.01^a^	2.293 ± 0.00^a^
	Beni Mellal	65.55 ± 0.01^b^	85.32 ± 0.08^b^	1.47 ± 0.01^a^	2.525 ± 0.01^a^
	Berkane	42.71 ± 0.04^c^	65.47 ± 0.14^a^	1.855 ± 0.00^b^	1.401 ± 0.00^b^
Seed	Settat	888.29 ± 0.02^d^	4039.88 ± 0.02^c^	0.020 ± 0.00^c^	ND
	Beni Mellal	1945.13 ± 0.03^e^	4832.78 ± 0.12^d^	0.019 ± 0.00^c^	ND
	Berkane	1333.22 ± 0.19^f^	4215.56 ± 0.08^e^	0.012 ± 0.00^d^	ND

Different letters of within the same column is indicating significant differences at *p* < 0.05. All data are expressed as mean ± SD of at least three replicates of each sample. dw: dry weight; AA: ascorbic acid.

**Table 3:** Correlation between polyphenol compositions antioxidant capacities.

	Total polyphenols	Total flavonoids	Punicalagin-*α*	Punicalagin-*β*	Ellagic acid	Gallic acid	DPPH assay	ABTS assay	Fe^2+^ chelating activity	FRAP assay
Total polyphenols	1.00									
Total flavonoids	.987	1.00								
Punicalagin-*α*	.986	.972	1.00							
Punicalagin-*β*	.977	.964	.998	1.00						
Ellagic acid	.995	.988	.994	.987	1.00					
Gallic acid	.979	.954	.996	.992	.987	1.00				
DPPH assay	-.908	-.892	-.908	-.906	-.908	-.906	1.00			
ABTS assay	-.987	-.975	-.992	-.988	-.992	-.988	.949	1.00		
Fe^2+^ chelating activity	-.977	-.966	-.982	-.978	-.982	-.981	.947	.995	1.00	
FRAP	.966	.946	.994	.998	.977	.993	-.898	-.981	-.972	1.00

The results are expressed as Pearson's coefficient (*p* value).

## Data Availability

The data used to support the findings of this study are included within the article.
